# Muscle Performance Investigated With a Novel Smart Compression Garment Based on Pressure Sensor Force Myography and Its Validation Against EMG

**DOI:** 10.3389/fphys.2018.00408

**Published:** 2018-04-19

**Authors:** Aaron Belbasis, Franz Konstantin Fuss

**Affiliations:** ^1^School of Engineering, RMIT University, Melbourne, VIC, Australia; ^2^Smart Equipment Engineering and Wearable Technology Program, Centre for Design Innovation, Swinburne University of Technology, Melbourne, VIC, Australia

**Keywords:** smart compression garment, force myography, pressure sensors, EMG, cycling, crank polar diagram, muscle fatigue, fractal dimension

## Abstract

Muscle activity and fatigue performance parameters were obtained and compared between both a smart compression garment and the gold-standard, a surface electromyography (EMG) system during high-speed cycling in seven participants. The smart compression garment, based on force myography (FMG), comprised of integrated pressure sensors that were sandwiched between skin and garment, located on five thigh muscles. The muscle activity was assessed by means of crank cycle diagrams (polar plots) that displayed the muscle activity relative to the crank cycle. The fatigue was assessed by means of the median frequency of the power spectrum of the EMG signal; the fractal dimension (FD) of the EMG signal; and the FD of the pressure signal. The smart compression garment returned performance parameters (muscle activity and fatigue) comparable to the surface EMG. The major differences were that the EMG measured the electrical activity, whereas the pressure sensor measured the mechanical activity. As such, there was a phase shift between electrical and mechanical signals, with the electrical signals preceding the mechanical counterparts in most cases. This is specifically pronounced in high-speed cycling. The fatigue trend over the duration of the cycling exercise was clearly reflected in the fatigue parameters (FDs and median frequency) obtained from pressure and EMG signals. The fatigue parameter of the pressure signal (FD) showed a higher time dependency (*R*^2^ = 0.84) compared to the EMG signal. This reflects that the pressure signal puts more emphasis on the fatigue as a function of time rather than on the origin of fatigue (e.g., peripheral or central fatigue). In light of the high-speed activity results, caution should be exerted when using data obtained from EMG for biomechanical models. In contrast to EMG data, activity data obtained from FMG are considered more appropriate and accurate as an input for biomechanical modeling as they truly reflect the mechanical muscle activity. In summary, the smart compression garment based on FMG is a valid alternative to EMG-garments and provides more accurate results at high-speed activity (avoiding the electro-mechanical delay), as well as clearly measures the progress of muscle fatigue over time.

## Introduction

*The European Parliament Scientific and Technology Options Assessment Panel … identified wearables as one of the ten technologies which will change our lives. Market prospects for wearables are very promising: wearables shipments are forecasted to increase to $150 billion by 2026 from the estimated level of $30 billion in 2016* (European Commission, 2016).

Wearable technologies were the most popular and leading fitness trend in 2016 for the first time, and continued to be so in 2017 (Thompson, 2015, 2016). The major drawback of smart wearables, in contrast to non-wearable laboratory equipment, is that their technology is not very accurate yet, mainly due to too many unvalidated products in the market (Düking et al., 2016).

This research deals with smart wearables for muscle performance assessment, the gold standard of which is undeniably electromyography (EMG). There are several problems associated with EMG, clearly pointed out by De Luca (1997) which makes it difficult to use EMG in wearables:

(1) EMG measures the electrical activity of a muscle which the mechanical activity lags behind (electro-mechanical delay).
(2) The amplitude of the EMG signal is non-linearly correlated to the muscle force, and depends on the number of motor segments recruited on the surface of the muscle, next to the electrodes.
(3) The electrodes should be located in the midline of the muscle, halfway between innervation zone and the next myotendinous junction.
(4) Shifting the electrodes along the action line of the muscle decreases the signal amplitude and a sideward shift decreases the amplitude of higher frequencies (thereby suggesting fatigue if the textile integrated electrode moves sideways).
(5) Tri-polar electrodes are preferable over bipolar ones, as the former eliminate crosstalk between muscles.

Furthermore, gel-/salt-based electrodes are required to reduce the skin resistance, although special design of embroidered electrodes can overcome this problem (Taelman et al., 2007; Shafti et al., 2017).

In spite of the issues pointed out above, two companies are selling EMG-based garments for performance analysis: Athos (Mad Apparel Inc., Redwood City, CA, United States) and Myontec (Myontec Ltd., Kuopio, Finland). A third company, Leo (GestureLogic Inc., Ottawa, Canada), developed an EMG thigh-sleeve but never sold the product (Early, 2016). B10nix^1^ (B10NIX Ltd., Milano, Italy) have announced an EMG-based shirt that is not commercially available yet. Athos^2^, for example, assesses right-left muscle imbalance. Given the fact that precise electrode placement is crucial for accurate results, equal activity levels of muscle groups on the right and left side of the body would generate different signals if the electrode were not placed on the same spot on both right and left muscle groups. To the best knowledge of the authors, there is not a single research paper available on validation of the Athos garments, in contrast to Myontec garments (e.g., Finni et al., 2007).

There are some research papers available that investigate prototypes of EMG-based garments for activity analysis (Taelman et al., 2007; Finni et al., 2007; Ribas Manero et al., 2016; Shafti et al., 2017). Finni et al. (2007) used traditional EMG electrodes incorporated in a garment, whereas Shafti et al. (2017) utilized customized, embroidered electrodes, validated with traditional gel-electrodes. Taelman et al. (2007) investigated the effect of electrode misalignment in a smart shirt, in the same way as Belbasis et al. (2015a) did (cf. Figure 1 of Belbasis et al., 2015a). Ribas Manero et al. (2016), however, did not validate their leggings prototype.

De Luca (1984) was the first to develop the concept of myoelectrical manifestations of localized muscle fatigue (Merletti et al., 1990). Fatigue is expressed in the EMG signal as an increase in EMG amplitude (increase of motor unit recruitment or synchronization by the central nervous system to maintain the required force level, related to *central fatigue*) and a shift to the lower frequencies of the EMG frequency spectrum (decrease of the conduction velocity of motor unit action potentials over the muscle, related to *peripheral fatigue*) (Mesin et al., 2009; Crozara et al., 2015).

The Myontec garment measures the muscle fatigue threshold (EMG_FT_2 according to Crozara et al., 2015), i.e., breakpoint in the linear relationship between EMG amplitude and exercise intensity (Lucia et al., 1999). The muscle fatigue threshold, however, is not suitable for measuring the increasing fatigue over time. Ribas Manero et al., 2016 were the first that attempted to measure fatigue with an EMG garment prototype, by using the *instantaneous Average Rectified Value* (iARV) signal. However, they did not validate the fatigue data they obtained. For example, although their iARV signal is supposed to increase with fatigue, their initial data at the beginning of the exercise are also very high. Another limitation in this technique is that sweat increases the iARV signal (Ribas Manero et al., 2016).

There are several methods available for the assessment of fatigue with EMG, such as FFT-based, time-based, amplitude-based, and wavelet-analysis-based methods. Details can be found in comprehensive reviews of Cifrek et al. (2009) and Gonzalez-Izal et al. (2012). Both papers mention fractal dimension (FD) methods without going into detail. The most common method for assessment of fatigue (gold-standard method) is FFT-based, and the onset of fatigue is characterized by a shift of the median frequency to smaller frequencies (De Luca, 1997). Basmajian and De Luca (1985) conducted an isometric experiment that shows the difference between mechanical fatigue and metabolic fatigue (measured with EMG and FFT method): the muscle force decreased at the failure point, whereas the preceding fatigue point was only detectable with EMG through the decreasing median frequency (see Figure 8.1 in Basmajian and De Luca, 1985).

The FD methods for assessing muscle fatigue have increased in importance over the last 10 years, with researchers using different methods, such as the box-counting method (Troiano et al., 2008, Beretta-Piccoli et al., 2015; Boccia et al., 2016) to understand the fractal behavior. Marri and Swaminathan (2016) used several methods [e.g., Higuchi (1988), Katz, Sevcik, box counting; multifractal analysis]. In most cases, Marri and Swaminathan’s (2016) monofractal algorithms delivered smaller FDs for fatigued muscles compared to non-fatigue; while the opposite was true for multifractal algorithms where the FD was mostly smaller than 1. In general, a signal’s FD ranges between a value of 1 and 2, i.e., between a straight line or smooth curve, and a maximally noisy signal filling up an area (Fuss, 2013).

Mesin et al. (2009) compared the FD of EMG signals to other muscle fatigue indexes, indicating that EMG FD was least affected by changes in conduction velocity and most related to the level of motor unit synchronization, and suggesting that the FD is an index of central rather than peripheral fatigue.

Furthermore, Mesin et al. (2009) found that in a power-trained subject, FD does not have a clear trend, indicating that the level of motor unit synchronization does not change, whereas the rate of change of the median frequency is high. In an endurance-trained subject, the rate of change of the median frequency is lower than in the power-trained subject, whereas rate of change of FD was high. These results suggest that power-trained athletes are affected more by peripheral fatigue, whereas endurance-trained athletes suffer more from central fatigue. Consequently, EMG-FD seems to be more sensitive in endurance-trained muscles, and EMG-FFT more sensitive in power-trained muscles regarding fatigue.

An alternative method to EMG is mechanomyography (MMG; Islam et al., 2013). In contrast to surface EMG, the quality of the MMG signal is not affected by electrical interference and changes of skin conditions as MMG measures the mechanical action of a muscle. MMG offers two methodological options:

(1) Vibromyography or acoustic-myogram (phono-myography) using accelerometers and/or microphones.
The method assesses the low amplitude sound of lateral oscillations generated by volumetric changes in active muscle fibers at frequencies between 5 and 100 Hz with microphones or low mass accelerometers (Fang et al., 2015). However, the signals are affected by limb movements and ambient noise, such that the method is not suitable for sports applications (Islam et al., 2013).
(2) Pressure sensors used for force myography (FMG). The sensors measure the pressure exerted by the muscles against the skin by volumetric changes of the active muscles (Castellini et al., 2014; Connan et al., 2016). Muscle bulging increases the pressure non-linearly with respect to the increase in muscle force (Belbasis et al., 2015a). The most common sensors used for FMG purposes are off-the-shelf FSR (force sensing resistive) sensors, either as single sensors, several sensors (Connan et al., 2016) or sensor matrix arrays (Zhou et al., 2017), that are preloaded, compressed either by tight fitting garments or by elastic bands to the surface of the relevant muscles (Lukowicz et al., 2006; McLaren et al., 2010; Zhou et al., 2017), Velcro bracelets (Connan et al., 2016), integrated in a textile sleeve (Ogris et al., 2007), equipped with mechanical preload adjustments (Li et al., 2012), or placed inside a forearm orthosis (Wininger et al., 2008). Belbasis and Fuss (2015) and Belbasis et al. (2015a,b) used several customized piezoresistive polymer sensors sandwiched between compression garment and skin. Meyer et al. (2006) applied a capacitance pressure sensor array embedded in textiles. Alternatively, Cheng et al. (2010) did not use any sensors but instead measured the body capacitance and its changes with movement.

The FMG or pressure sensor-based garments are a typical example of lateral innovation, i.e., achieving the same goal with other or alternative means, a common precursor of a disruptive technology. Lateral innovation is characterized by, e.g., lower costs, higher accuracy, better user-friendliness, smaller hardware, simpler solution, simpler implementation, less affected by error and method, better wearability, providing additional information, or improved manufacturability (Fuss, 2017). However, none of these FMG solutions are commercially available yet.

The aim of this paper was to explore an existing prototype of pressure sensor-based garment (Belbasis and Fuss, 2015; Belbasis et al., 2015a,b) for opportunities in performance analysis, specifically muscle activation and fatigue, and to validate the prototype against EMG, used as the gold standard for muscle performance assessment.

The method selected for this task had to comprise of a standardized repeatable activity and a defined fatigue protocol. We used cycling on a stationary power-controlled bicycle as the method of choice. Fatigue was assessed through the Fast Fourier Transform (FFT, gold standard) of the EMG signal, as well as with FD signal processing. For the latter, the Higuchi’s (1988) method is considered the gold standard method, however, a new customizable FD method (Fuss’ method; Fuss, 2013) was selected that offers advantages over Higuchi’s (1988) method.

## Materials and Methods

### Participants

Seven male participants (age: 28 ± 3.6 years; body height: 1.751 ± 0.059 m; body mass: 78.7 ± 7.9 kg) were involved in the experiments. This study was granted Ethics approval by the RMIT University Human Ethics Committee (approval no. ASEHAPP 45-15) and adhered to the Declaration of Helsinki. An informed consent form was filled in by all the participants before the start of the experiment.

All participants were deemed healthy volunteers, passing RMIT University Ethics Committee approval for health requirements to sustain the level of exertion required during the tests. The participants were all of above-average levels of fitness participating in various sports such as running (participant 1 and 5), soccer (2 and 4), and cycling (3, 6, and 7) at least three times a week. The overall cycling skill range was from Amateur (participant 2) through to Semi-elite (participants 3 and 7).

### Data Collection

**FIGURE 1:**
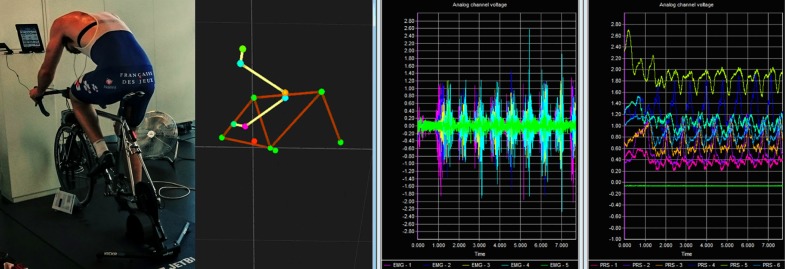
Experimental set-up, motion capture, EMG signal, and muscle pressure signals; the latter three subfigures are screen shots of the software; the unit of the EMG signal on the screen shot is mV⋅10^-2^ and unit of the pressure signal on the screen shot is V.

A motion capture system (9 Camera – Qualisys Oqus System, Göteborg, Sweden) was utilized to capture the limb segment angles of the participants, as well as providing tracking for the rotational crank angle of the bicycle (**Figure 1**). The data sampling frequency for motion tracking was set at 100 Hz, where the marker positions are shown in **Figure 1**.

A previously developed smart compression prototype garment (Belbasis and Fuss, 2015; Belbasis et al., 2015a,b) was utilized for the testing of each athlete. The garment provided capability for measuring and mapping changes in the surface pressure above a muscle (**Figure 1**) where the active movement of the muscle under the compression fabric was detected by a distributed network of pressure sensors. The low-pressure sensors were manufactured from two layers of a conductive piezoresistive polymer, with an almost linear calibration curve of the average equation of *P* = 97282000 σ^1.184335^ for two layers, where *P* is the pressure in Pascal, and σ is the conductivity in Siemens (Fuss et al., 2016).

The sensors were positioned over five of the thigh muscles [rectus femoris (RF), vastus medialis (VM) and vastus lateralis (VL), biceps femoris (BF), and semitendinosus (ST)] of the participant’s right leg. In addition to the utilization of pressure sensors, a 16-channel wireless EMG system (Wave Plus Wireless EMG, Cometa Systems, Bareggio, Italy) was used for recording the electrical signal (**Figure 1**) of the same muscles. The general placement of the electrodes followed the recommendations of SENIAM [Surface Emg for NonInvasive Assessment of Muscles] (1999) and the optimum placement of the electrodes was achieved by using the method of Belbasis et al. (2015a). To ensure accurate capture of the muscle behavior throughout the tests a data sampling frequency of 2000 Hz was utilized for both the pressure and EMG sensors.

### Experimental Method

A fatigue-inducing regiment based upon work by Dorel et al. (2009) was developed to quantify the effects of fatigue during cycling. The test protocol deliberately introduced fatigue to the active muscles, allowing for the analysis of muscle activity and performance under two known definitive conditions, namely, a non-fatigued and a fatigued state. To allow for sufficient muscle recovery, participants were asked to follow the following testing procedures over two testing sessions which were separated by at least 4 recovery days.

The tests were performed on the participant’s own bicycle mounted on the stationary ergometer (Wahoo Kickr, Wahoo Fitness, United States).

To ensure that muscles were activated during the upstroke of the pedal phase (180–360° of the crank cycle) clip-in shoe/pedal combinations or caged pedals were utilized to prevent separation of the foot and pedal.

The test persons performed a cycling exercise at a constant power output equal to 80% of their functional threshold power (FTP) for as long as possible; and maintained a constant pedaling rate (cadence). The test continued until the cyclists were no longer able to maintain their initial test cadence (±5 rpm).

#### Session One: FTP Ramp Test

Each participant was tasked with completing an incremental cycling exercise (Ramp test). This involved the incremental ramp-up of generated power to determine the exercise limitations of the participant. Other than a heart-rate strap, no instrumentation of the participant’s body was necessary for this session. All testing begun at a target power output of 120 Watts with increasing workload increments of 20 W/min until the target power could no longer be satisfactorily sustained.

To ensure consistent power output during the test the ERG-mode setting of the Wahoo Kickr ergometer was utilized. This setting constantly monitors the generated power and cadence (angular velocity), and enforces a consistent target power output through automatic adjustments to the cycling resistance level (torque) through a magnetic actuator.

To prevent artificially enforcing an earlier end to the test, reasonable changes in both cadence and gearing were permitted by the participant to find their comfort zone to complete the task. The FTP, defined as the last stage that was completed in its entirety, was used to calculate the appropriate workload imposed by the cycle ergometer during the second test session.

#### Session Two: 80% FTP Fatigue Test

The second session, notably the primary data collection session, involved the complete instrumentation of the participant’s right upper leg with EMG, motion capture, and pressure sensor equipment. Participants performed a self-directed warm-up routine consisting of at least 3 min of cycling at a lower power output to the test condition, ensuring sufficient preparation of the participant for the test. Following the warm-up, subjects performed a cycling exercise at a constant power output equating to 80% of their measured FTP for as long as physically maintainable. The ergometer was set at a fixed resistance setting and the participant instructed to maintain the two target parameters displayed to them; the target power output (80% FTP), and a constant cadence freely adopted from the end of the warm-up session. Surface muscle pressure, EMG and angular parameters were recorded continuously throughout the session.

To enforce repetitive muscle activation, participants were asked to maintain a single cycling position, where shifting along the saddle or handlebars was not allowed. The test continued until the cyclists voluntarily chose to stop the exercise (fatigue-induced exhaustion) or until they were no longer able to maintain their initial test cadence (± 5 rpm), which was considered as a failure to maintain the required task (the target power output at a constant cadence).

### Data Analysis and Statistics

The raw data of both pressure and EMG signals were recorded in volts and millivolts, respectively, at a frequency of 2000 Hz, simultaneously and synchronized with the motion capture data utilizing a centralized trigger device. From the pedal marker, the top dead center of the crank (highest marker position) was set to 0° with increases in crank angle in the clockwise direction (as viewed from right-hand side of the bicycle).

For the muscle activity analysis, the signal amplitude (of pressure signal and EMG) for ± 1.5 SD (removal of outliers) was assigned to the crank angle. The average amplitude was calculated with a running median filter of a window width of 7.5°.

Subsequently, the average crank cycle data were normalized. In order to calculate the average signal of each muscle across all seven participants, the data of all participants were averaged, squared (thereby assigning a greater weight to higher data), and normalized again. The average crank angle of each muscle was determined from that angle that divides the areas under the signal into two equal parts (integration window = 180°). The average crank angle represents the position of the activated muscle on the crank diagram as a single number for comparative purposes.

For the fatigue analysis, the raw signal amplitude was expressed as a time series with a fourth-order Butterworth bandpass filter (10–350 Hz) applied to the EMG data to remove noise. Raw pressure values were utilized with no further filters applied, however, the original sampling frequency was reduced to 80 Hz via postprocessing, due to the smoothness of the pressure signals. Each of the muscle signals were subjected to FFT (EMG only) and fractal dimensional analysis (EMG and pressure signal). De Luca (1997) established that the median frequency of an EMG signal over a set time period shifted toward lower frequencies as a result of increasing muscular fatigue. The negative trend of the median frequency over time provided an understanding of the performance decrease in the muscle under investigation. More specifically to cycling and this research, the analysis builds on the approach taken by Dingwell et al. (2008) by utilizing a Short-Time Fourier Transform (STFT) technique, whereas the calculation of the power spectrum, and the resultant median frequency, is performed over individual time segments attributed to each crank cycle revolution. All calculation was made using the FFT function within MATLAB (The MathWorks, Inc., Natick, MA, United States) and a sliding average window of 1 min width to define the averaged trend of the data.

**FIGURE 2:**
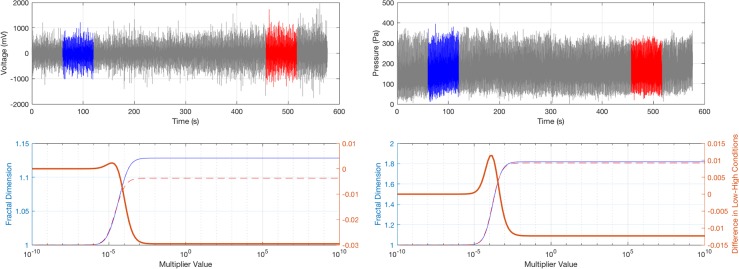
Fractal dimension (FD) optimization procedure (Fuss, 2013); (Left) EMG; (Right) pressure; top row: raw data and data segments used for calculating the FD differential of fresh (blue) and fatigued (red) muscle; bottom row: FDs and FD differential against multiplier of signal amplitude; blue curve: FD of fresh muscle; dashed red curve: FD of fatigued muscle; bold orange curve: FD differential (FD of fatigued muscle – FD of fresh muscle); the optimal multiplier of signal amplitude is found at the maximum (peak) of the bold orange curve.

The FD of EMG and pressure signals was calculated with the method developed by Fuss (2013). This method allows for maximal separation of two conditions (e.g., fresh and fatigued muscle states) by means of adjusting and optimizing the signal amplitude multiplier. If this multiplier is set to high values (infinity in theory), then Fuss’ method is identical to Higuchi’s (1988) method. In order to identify the optimal amplitude multiplier, the EMG and pressure signal’s FDs were calculated for the second (fresh muscle) and second last (fatigued muscle) full minute of the tests at different multipliers. The differential of the FDs of fatigued and fresh states (**Figure 2**) was plotted against the decadic logarithm of the multiplier (Fuss, 2013) and the optimal multiplier was identified at the maximum differential. This amplitude multiplier was then used to calculate the FDs continuously through the signals with a running window width of 1 min.

Both median frequency data and FDs data were normalized. For comparing the fatigue development across all participants, the time was normalized as well (due to different experiment durations; *cf*. **Table 2**). The median frequency data and FDs data were linearly correlated to the normalized time to asses**s** the percentage of the time dependence by means of *R*^2^. The *R*^2^-values were compared as to their significant difference with Fisher’s *Z*-test for comparing correlations from independent samples.

## Results

### Power Data

The primary objective of the first testing session was to determine each participant’s achievable FTP wattage level, allowing for the normalized testing FTP target during the second test. Outputs from the cycling trainer pertaining to the participant’s performance data were collected and are shown in **Table 1**. Application of the ramp test specifically assessed an individual’s ability to increasingly deliver higher power output over time, as such we expect a distribution in the resultant efforts throughout the sample group because of differences in physical ability and familiarization with the task. Due to the similarity in skill set and fitness between the participants, five participants fell within the bounds of one SD from the mean of the duration and achieved FTP level. The other two participants, namely, the least experienced cyclist (participant 2; **Table 1**), and the most experienced (participant 3) were within two SDs.

**Table 1:** Session one activity summary.

Participant	Duration (min:s)	Duration (s)	FTP Level (W)	Total work (kJ)	Mean cadence (rpm)	Mean cadence (rad/s)	Mean power (W)	Mean torque (Nm)
1	10:35	635	320	141	88	9.2	222	24.1
2	06:09	369	220	62	58	6.1	168	27.7
3	17:11	1031	420	272	82	8.6	264	30.7
4	12:12	732	320	151	74	7.7	206	26.6
5	09:11	551	260	100	70	7.3	181	24.8
6	12:14	734	340	167	90	9.4	228	24.1
7	13:07	787	360	189	68	7.1	240	33.7
Mean	11:31	691	320	154.57	75.71	7.9	216	27.4
*SD*	03:26	206	65.32	66.92	11.57	1.2	33	3.7

*FTP = functional threshold power, i.e., the maximal power achieved in the incremental ramp-up of generated power (stepwise increase of power starting at 120 W)*.

Following the determination of the participant’s FTP level, individual 80% FTP calculations were made for each participant and utilized for the second session to ascertain fatigue behavior. This inclusion of the additional biomechanical measurement systems (Pressure, EMG, and MOCAP) within the second test session allowed for greater insight into the onset and continued fatigue behavior of the muscles in the lower limbs.

A summary of key test data relating to each test is shown in **Table 2**. Accuracy of achieving the target of 80% FTP loading required was met within a satisfactory range (5%) for each participant with the mean accuracy within 1% of the grouped aim.

**Table 2:** Session two activity summary.

Participant	Duration (min:s)	Duration (s)	Mean power (W)	Total work (kJ)	Mean cadence (rpm)	Mean cadence (rad/s)	Mean torque (Nm)	Target Power (W)	Target Accuracy %
1	09:28	568	259	147	84	8.8	29.4	256	98.84
2	03:28	208	175	36	68	7.1	24.6	176	100.57
3	11:18	678	330	224	81	8.5	38.9	336	101.82
4	09:15	555	257	143	71	7.4	34.6	256	99.61
5	12:41	761	208	158	64	6.7	31.0	208	100
6	09:52	592	268	159	80	8.4	32.0	272	101.49
7	12:00	720	275	198	66	6.9	39.8	288	104.73
Mean	09:43	583	253	152.00	73	7.7	32.9	256	101.01
*SD*	03:03	183	49.6	59.1	8.1	0.8	5.4	52.3	1.94

*Target power = 80% of FTP shown in **Table 1**; target accuracy = target power/mean power × 100*.

A noticeable deviation in the results was the duration of the test for participant 2 (least experienced). While all other participants concluded the test within one SD of the test mean (9:33 min of exercise), the fatigue tolerance for participant 2 forced an end to the test after only 3:28 min. This result aligns with the experience level of the participant in comparison with that of the other participants, where duration of the test is largely driven on the physiological and psychological conditioned nature of the muscle and participant to operate under increasing fatigue-limiting conditions. The experience level also correlated with the mean power and torque (**Table 2**) such that the least (participant 2) and most experienced (participants 3 and 7) participants exhibited the lowest and highest values, respectively.

### Muscle Activation

Through the motion capture of the pedal stroke movement, the muscle activity was resolved to the corresponding angle of the crank where each individual muscle was utilized, shown on polar diagrams.

**FIGURE 3:**
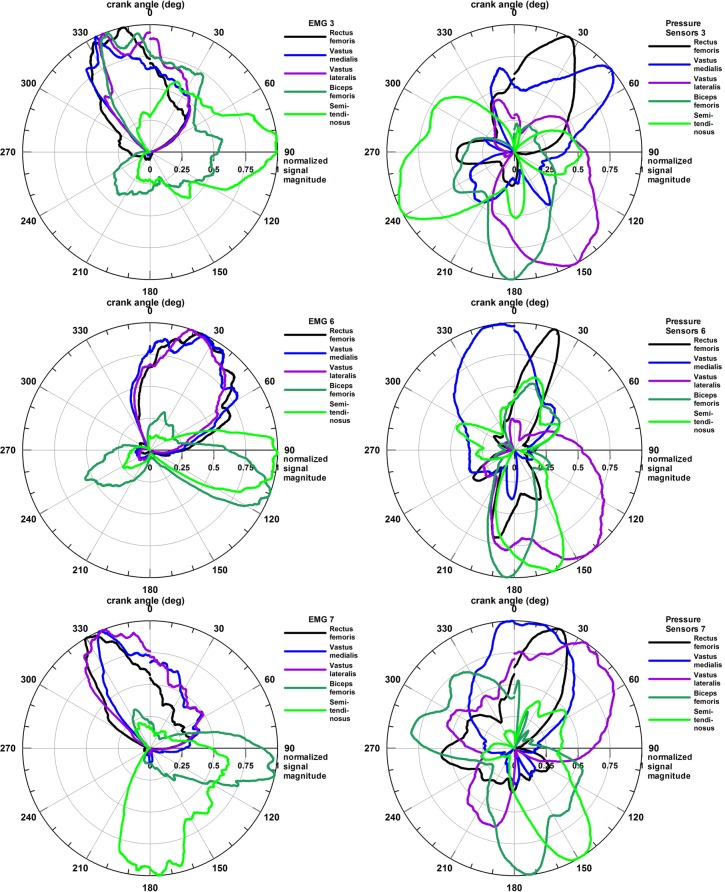
Polar plots of the activity of five muscles and three participants; Left column: EMG data, Right column: force myography data (pressure data).

The polar diagrams of three representative participants are shown in **Figure 3**. The EMG graphs of the extensors (RF, VM, and VL) exhibited overlapping activity in the same sector of the diagram, with individual differences: in **Figure 3** (top and bottom rows) at 330–360°, whereas in **Figure 3** (middle row) at 30°. The pressure-based activity deviated from the EMG-based activity in general by a clock-wise phase shift. For example, in **Figure 3** (bottom row), the extensors still overlap, although not that perfectly as in the EMG plot, but the peak activities are shifted by 30–60° clockwise. In **Figure 3** (middle row), RF shows pressure and EMG activity in the same sector, whereas for VM, the pressure signal is shifted counter clockwise by approximately 30° with respect to the EMG signal, and VL is shifted clockwise by more than 60°. In **Figure 3** (top row), RF and VM are shifted clockwise by 30° and 70°, respectively, and VL by almost 180°.

Comparing the three pressure plots, the activity of RF ranges from 20 to 30°, VM from -10 to 50°, and VL from 40 to 150°.

The flexor muscles (BF and ST) showed less consistent EMG activation patterns than the extensors: ST at 90°, 90°, and 180°; and BF at 100°, 110°, and 340°. The pressure activation patterns are, in general, shifted clockwise as already seen in the extensor muscles, namely the BF by 70°, 70,° and 200°; and the ST by -30°, 70°, and 150°.

Comparing the three pressure plots, the peak activity of BF occurs around 170–180°, whereas the one of ST ranges from 150 to 240°.

**Figure 3** (top row) shows a co-contraction of the three extensors and the BF on the EMG plot, whereas the pressure plot confines the co-contraction to VL and BF. The same is true for both the hamstrings and the VL on the pressure plot [**Figure 3** (middle row)], whereas the EMG plot appears to be free of co-contractions. The latter is true for both pressure and EMG plots in **Figure 3** (bottom row).

**FIGURE 4:**
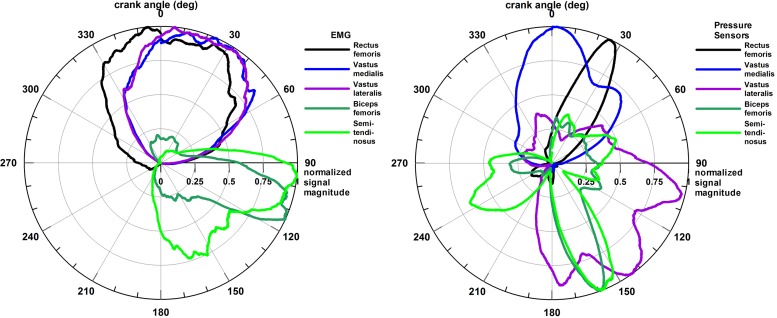
Combined polar plots of the activity of five muscles of all seven participants; Left plot: EMG data, Right plot: force myography data (pressure data).

**Figure 4** shows the average muscle activation patterns of all seven participants combined, thereby highlighting the sectors used by most participants.

In general, while the muscle activities, measured with EMG or pressure, are relatively consistent across athletes, they do not coincide when the two different methods are compared directly (**Figures 3**, **4**).

The average angles of the EMG signal are: RF – 8°, VM – 24°, VL – 23°, BF – 110°, and ST – 122°; and of the pressure signal of the five muscles are: RF – 24° (phase shift +16°), VM – 8° (phase shift -16°), VL – 124° (phase shift +101°), BF – 143° (phase shift +33°), and ST – 156° (phase shift +34°);

The pressure plots of all but one muscle are characterized by a clockwise phase shift with respect to the EMG plots of 16–101°. Only VM is shifted counter-clockwise by 16°. This phase shift phenomenon is attributed to the electromechanical delay of the muscle signal, which will be explained in detail in the section “Discussion.”

### Muscle Fatigue

Assessment of the fatigue performance over the entirety of the second test was made through two different measurement methods and two different algorithms resulting in the need to compare by correlation three different fatigue signals, namely, the FFT (FFT median frequency; FFT-EMG) and the FD (FD-EMG and FD-Pressure).

**FIGURE 5:**
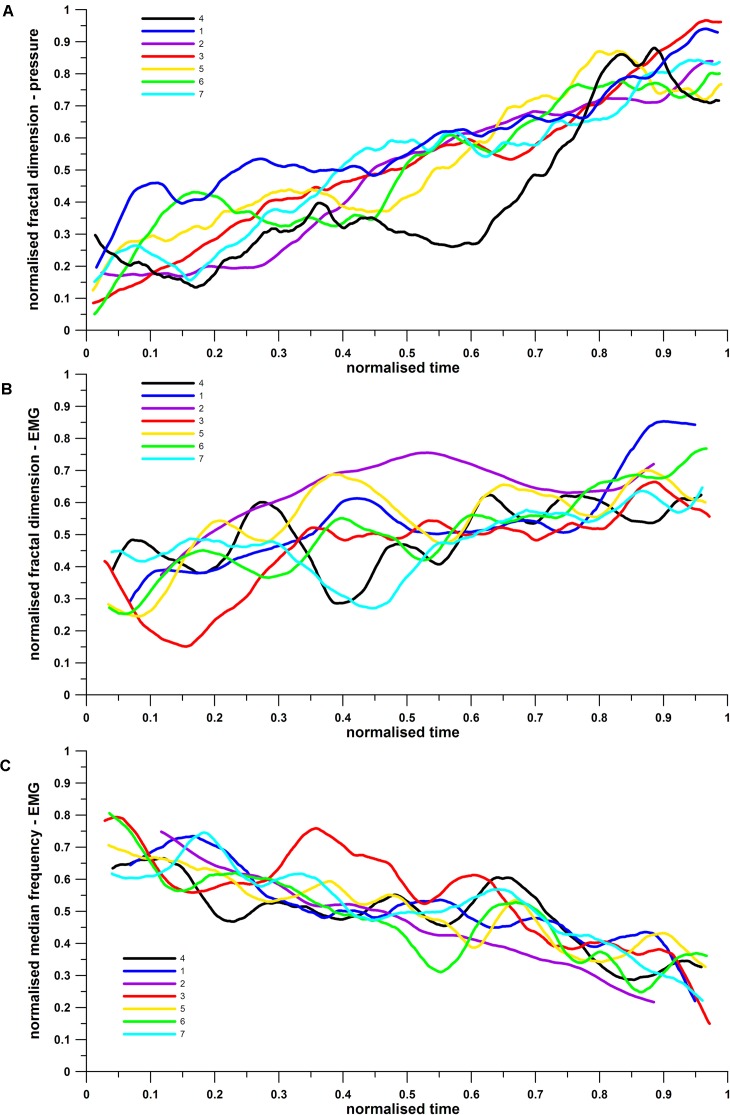
Normalized average fatigue (fractal dimensions and median frequency) vs. normalized time: **(A)** pressure fractal, **(B)** EMG fractal, and **(C)** EMG median frequency.

In general, when considering the overall behavior of each participant (**Figure 5**), the overall fatigue trend is clearly seen in all signals, with increasing (fractals) and decreasing (FFT) trends.

The normalized pressure fractals correlate with the normalized cycling time in 84% of the results (*R*^2^ = 0.8405, linear fit; 84% of the fatigue level is explained from the time progression of the exercise). The normalized EMG fractals and median frequencies correlate with the normalized cycling time in 51% (*R*^2^ = 0.5081) and 71% (*R*^2^ = 0.7092) of the results respectively. All three *R*^2^ values are significantly different (*p* = 0).

The *R*^2^-value expresses merely that, for the FFT method, 71% of the fatigue level are time-dependent whereas 29% are not time-dependent. Time-independent fatigue would be if a fatigue level or the average fatigue was kept relatively constant over a longer time. Furthermore, the different performance levels of the subjects could also contribute to the time-independent fatigue; for example, more experienced athletes are more skilled in fatigue management over time. FD-EMG reflects more time independent fatigue (49%) compared to FD-pressure (16%), i.e., approximately three times as much. This phenomenon will be discussed in more detail in the section “Discussion.”

## Discussion

The purpose of this study was to explore the applicability of a smart compression garment based on FMG with pressure sensors (Belbasis and Fuss, 2015; Belbasis et al., 2015a,b), measuring muscle contraction, for assessment of muscle activity and fatigue, as an alternative to EMG.

First of all, it is worth noting, that while the maturity of the pressure monitoring technique is still in development, the majority of common experimental issues (such as restarts, corrupted data, time-consuming instrumentation of athletes) was attributed to the installation and attachment of the EMG equipment and electrodes, and the motion capture markers. The simplicity and robustness of the wearable smart compression garment system limited the possibility of experimental failures.

The first question to address is whether muscle activity can be assessed and measured with the smart compression garment. The signals obtained, related to the contraction pattern when cycling, were highly comparable and consistent on the polar diagrams, with some individual differences between participants.

The second objective of this study was to validate the muscle activity pattern obtained from the smart compression garment with a gold standard, i.e., a laboratory-based EMG system. However, the muscle activation patterns obtained from EMG and the smart compression garment were, to some extent, not comparable (**Figures 3**, **4**). The reason for this is not the inferiority of the smart compression garment, which could be easily deduced from the data, but rather the choice of the gold standard. Undoubtedly, EMG is the (even if the only) gold standard for assessment of muscle activity and fatigue. Yet, EMG measures the electrical activity of the muscle, whereas the smart compression garment detects the mechanical activity, i.e., muscle bulging that compresses the pressure sensors between skin and garment. The difference between EMG and pressure-sensor polar plots simply reflects the difference between electrical and mechanical activity. The electro-mechanical delay (De Luca, 1997) of the contraction force with respect to the electrical stimulation of a muscle is explained from the time difference between onset of electrical activity and the increasing muscle force. This delay is also dependent on muscle fiber distribution, i.e., the percentage of fast- and slow-twitch fibers. For example, to reach a contraction level of 50% of the maximal muscle force, it takes a fast- and slow-twitch fiber approximately 0.15 and 0.25 s, respectively (De Luca, 1997). When cycling at a cadence of 73 rpm (average cadence from **Table 2**), these two delay times would cause, in theory, a phase shift of 66° and 110° on the polar diagram. The differences seen in the EMG and pressure sensor polar diagrams are therefore expected. According to EMG data of Jorge and Hull (1986) and Hug et al. (2010), the quadriceps is active from 300 to 130° and from 235 to 162°, respectively, and the hamstrings from 15 to 255° and from 324 to 288°, respectively (maximal ranges). The data seen in **Figure 4** perfectly fit into these ranges, which the exception of the VL, which exceeds 130°. Jorge and Hull (1986) also reference other papers, the results of which show considerable differences and fluctuations, suggesting that there is considerable variety of EMG results.

Nevertheless, EMG is still a gold standard for validating the smart garment, as there is no other system available. The gold standard therefore serves primarily for understanding the differences between the data, and the underlying principles of the different measurement systems. Validation is still possible, if differences are known in the first place or at least expected, and subsequently confirmed through a validation study. This issue poses a new challenge for wearable technology not experienced before, specifically when dealing with lateral innovation (Fuss, 2017). Finding a suitable gold standard could then become a problem.

The third objective of this study was to assess whether muscle fatigue can be measured from the pressure signals. The evaluation was based on the calculation of the FDs of pressure and EMG signals. For calculating these FDs, Fuss’ method was used as it maximally separates the FDs of a normal and an abnormal signal, by finding the maximum differential of FD-abnormal - FD-normal, when subjecting both signals to the same amplitude multiplier. Normal and abnormal signals could be physiological/pathological ones, less/more chaotic ones, signals from fresh and fatigued states, low/high activity signals, etc. From common sense, the abnormal signal is expected to have a higher FD. Common sense is confirmed if there is a maximum differential, and the two asymptotic fractal differentials at multipliers of close to 0 or to infinity (**Figure 2**) are smaller than the maximum. It has been seen on numerous occasions, that Higuchi’s (1988) method, corresponding to Fuss’ method with an infinite multiplier, returns higher FD for normal signals (Fuss, 2013, 2016), compared to abnormal ones. This problem is seen in **Figure 2** as well, more pronounced in the EMG FD data, though. This behavior is not unexpected in the EMG signal, as the decreased amplitude of high frequencies in the power spectrum (typically seen in fatigued muscles) leads to a decrease of FD. The increase in EMG amplitude, also typical for fatigue, increases the FD. If the cadence drops, so does the FD. Even if there are multiple influences that affect the FD, it would be more logical to assume that the FD of a fatigued muscle’s signal is smaller than the one of a fresh muscle, if the principle of left-shift of the median frequency is known.

Irrespective of logical assumptions, all three methods applied, FD-Pressure, FD-EMG, and FFT-EMG, showed the same clear trend, namely, that fatigue increases with time, with some individual differences between participants.

The fourth objective of this study was to validate the muscle fatigue trend obtained from the smart compression garment with a gold standard, i.e., a laboratory-based EMG system. The same gold-standard problem as seen in the muscle activation patterns is also applicable to fatigue to some extent. When comparing FD-Pressure and FD-EMG to FFT-EMG, all three variables correlated to the normalized time of the experiments, FD-pressure showed highest time dependent correlation (84%), and FD-EMG the highest time-independent component (41%). These differences come from the fact that FD-Pressure is more related to mechanical fatigue, whereas FD-EMG and FFT-EMG are related to central and peripheral fatigue, respectively.

There is indication (Mesin et al., 2009) that shift of the median frequency of the EMG signal is related to peripheral muscle fatigue (decrease in conduction velocity) whereas the FD of the EMG signal is related to central fatigue (increase in motor unit synchronization). This seems illogical at first sight, as the higher the amplitude of higher frequencies is, the greater is the FD, and therefore any reduction of median frequencies is coupled to a smaller FD. This principle can be easily verified when using synthetic fractal signals, such as Knopp/Takagi function, Weierstrass cosine and Weierstrass-Mandelbrot functions, and stochastic Brownian Motion function (Fuss, 2013). However, EMG data are not based on functions that generate signals with predefined FDs. As such, low median frequencies and small FD do not necessarily exhibit a parallel trend. This possibility is also affected by the method used for calculating FDs.

Furthermore, there is indication that a power-trained subject was more affected by peripheral fatigue whereas an endurance-trained subject was more prone to central fatigue (Mesin et al., 2009). It is therefore expected that the correlation of fatigue parameters that measure different components of fatigue is not necessarily high. This correlation is not just affected by the fatigue component, but also by the distribution of training type across the participants of a study. For example, participant 3 is a long-distance cyclist and therefore endurance-trained, whereas participant 4 is a soccer player and thus power-trained.

If FD-EMG and FFT-EMG are related to central and peripheral fatigue, respectively, then FD-pressure could be related to mechanical fatigue. Mechanical fatigue is actually defined as the failure of the muscle system, i.e., that the force level cannot be maintained anymore (Basmajian and De Luca, 1985). Nevertheless, metabolic fatigue (measured with EMG) becomes apparent even before system failure (Basmajian and De Luca, 1985). As such, the term mechanical fatigue is probably not appropriate, and should be replaced by mechanical pre-fatigue.

## Conclusion

The smart compression garment based on FMG with pressure sensors returned performance parameters (muscle activity and fatigue) comparable to the surface EMG, used as gold standard for validation. The major differences were that the EMG measured the electrical activity whereas the pressure sensor measured the mechanical activity. As such, there was a phase shift between electrical and mechanical signals, with the electrical ones preceding the mechanical ones in most cases. This is specifically important in high-speed cycling, the activity investigated in this study. Using the activity sectors on the polar diagrams, obtained from EMG, for biomechanical models, could result in incorrect outcomes, compared to using the activity data obtained from FMG. The latter are considered more appropriate as input for biomechanical modeling.

In terms of fatigue, apart from individual differences between the participants, the fatigue trend over the duration of the cycling exercise was clearly reflected in the fatigue parameters (FDs and median frequency) obtained from pressure and EMG signals. The fatigue parameter of the pressure signal (FD) showed a higher time dependency (*R*^2^ = 0.84) compared to the EMG signal. This reflects that the pressure signal puts more emphasis on the fatigue as a function of time rather than on the origin of fatigue (peripheral or central).

## Author Contributions

AB and FF contributed equally to the design of the study, execution of the experiment, data analyses, and writing and editing the manuscript.

## Conflict of Interest Statement

The authors declare that the research was conducted in the absence of any commercial or financial relationships that could be construed as a potential conflict of interest.

## Footnotes

1 http://wise.b10nix.com/

2 https://www.liveathos.com/athletes
